# Is Boiling Bitter Greens a Legacy of Ancient Crete? Contemporary Foraging in the Minoan Refugium of the Lasithi Plateau

**DOI:** 10.3390/foods13223588

**Published:** 2024-11-10

**Authors:** Mousaab Alrhmoun, Naji Sulaiman, Shiekh Marifatul Haq, Syed Abidullah, Julia Prakofjewa, Nikos Krigas, Andrea Pieroni, Renata Sõukand

**Affiliations:** 1 Faculty of Agricultural, Environmental and Food Sciences, Free University of Bolzano, Piazza Università 5, 39100 Bolzano, Italy; 2 University of Gastronomic Sciences, Piazza Vittorio Emanuele II, 9, 12042 Pollenzo, Italy; 3 Department of Ethnobotany, Institute of Botany, Ilia State University, 0162 Tbilisi, Georgia; 4 Department of Botany Abdul, Wali Khan University, Khyber Pakhtunkhwa, Mardan 23200, Pakistan; 5 Department of Environmental Sciences, Informatics, and Statistics, Ca’ Foscari University of Venice, Via Torino 155, 30174 Venezia, Italyrenata.soukand@unive.it (R.S.); 6 Institute of Plant Breeding and Genetic Resources, Hellenic Agricultural Organization Demeter (ELGO DIMITRA), 57001 Thessaloniki, Greece; nkrigas@elgo.gr; 7 Department of Viticulture, Floriculture & Plant Protection, Institute of Olive Tree, Subtropical Crops and Viticulture, Hellenic Agricultural Organization Demeter (ELGO DIMITRA), 71307 Heraklion, Greece; 8 Department of Medical Analysis, Tishk International University, Erbil 44001, Iraq

**Keywords:** cooking processes, Crete, cross-cultural comparison, ethnobotany, Mediterranean diet, wild greens

## Abstract

Wild greens (WGs) play a significant role in Mediterranean diets (MDs), reflecting botanical and cultural diversities, mainly influenced by a complex conglomerate of local human ecologies. This study investigates local ecological knowledge (LEK) linked to traditional gathering and consumption of WGs in the Lasithi Plateau of eastern Crete, where human genetic studies one decade ago showed very peculiar patterns, hypothesising that the Minoan civilisation took refuge there before it disappeared. A field ethnobotanical study was conducted to document the diversity of WGs and their detailed local culinary uses in the Lasithi area by interviewing 31 study participants. Fifty-nine folk taxa (species and subspecies) were recorded, corresponding to fifty-eight botanical taxa. A quotation index was measured to assess the cultural significance of WGs in the study areas; logistic regression analysis was adopted to understand the impact of sensory classifications of WGs and their local cooking methods. Lasithi’s foraging showed a notable prevalence of bitter-tasting WGs, which play a central role in local cognition and culinary practices. This bitterness aspect of WGs, potentially influenced by cultural preferences and genetic factors, probably suggests a connection to the ancient Lasithi’s inhabitants, i.e., Minoan dietary habits. We found that bitterness is the predominant sensory attribute in Lasithi, characterising 45.76% of the WGs. These findings underscore the complex interplay between local ecologies and biodiversity, LEK, and dietary traditions, highlighting the importance of WGs in understanding the evolution of foraging and plant culinary diversities across the Mediterranean.

## 1. Introduction

Human health results from hereditary factors and environmental influences, notably nutrition [1]; on the other hand, food is a lifelong necessity, making nutrition a crucial ecological factor affecting human health [2]. The Mediterranean diet (MD) has garnered significant attention since its initial conceptualisation in the landmark “Seven Countries Study”, where its potential health benefits and cultural significance were highlighted [3,4]. MDs are characterised by consuming diverse fresh fruits, vegetables (many growing wild), whole grains, olive oil, and moderate amounts of dairy, fish, and poultry. This dietary pattern has not only been associated with reduced risks of chronic diseases but also reflects the Mediterranean region’s rich culinary traditions and ecological diversity [5]. An essential number of MD vegetables are characterised by bitterness, pungency, or both, and studies at the molecular level have established a rather direct link between compounds with these sensory properties and beneficial effects on health [6,7].

The Mediterranean region, renowned for its rich biodiversity and cultural heritage, encompasses diverse landscapes that shape wild greens’ (WGs’) availability across its territories and ecologies [8]. However, the intensity and exact typology of WGs’ traditional foraging and consumption reflect human ecological dynamics, cultural practices, and, possibly, genetic issues. For example, in a recent cross-comparative analysis of Mediterranean and Middle Eastern plant foraging, Armenians seem to consume fewer bitter WGs [9]; additionally, a recent genetic study has confirmed a higher proportion of super-testers among Armenians [10] and, therefore, possibly the prevalence of an innate inability to find palatable most bitter wild plants.

Across these diverse regions of the Mediterranean, wild greens play a pivotal role in culinary practices deeply rooted in local culinary histories and environments [11,12]. Crete’s secluded highland Lasithi Plateau has not been distinguished by its fertile lands and agricultural traditions but by its intriguing genetic peculiarity. The island of Crete, credited by some historical scholars as a central crucible of Western civilisation, has been under continuous archaeological investigation since the second half of the nineteenth century. More recently, genetic studies have further enriched archaeological research, reporting that the Lasithi area’s current inhabitants have a distinct genetic profile compared to all other Cretans and, most possibly, that this area could have represented the last Minoan refugium [13].

Crete stands out in the European context for its vast diversity of widely practised traditional WG foraging [11]. The starting idea of this work was to evaluate whether and how Lasithi’s historical and genetic specificities also reverberate in its traditional WG foraging. In Crete, wild greens are still regularly harvested and traded in shops or open markets alongside their cultivated counterparts [11]. Crete emerges as a focal point where agricultural practices have sustained traditional knowledge and culinary customs associated with wild greens. With a substantial portion of its population engaged in farming, Crete epitomises continuity in rural life and a deep-seated connection to the land [14,15].

The culinary uses of wild greens may vary widely, influenced by factors such as local biodiversity, historical agricultural practices, and societal preferences [16,17,18,19,20]. Studies exploring the culinary uses of wild greens have underlined their importance in traditional diets worldwide. For instance, Sicily, a biodiversity hotspot, exhibits various wild vegetable species used in local cuisines, reflecting centuries of culinary evolution influenced by Greek, Roman, Arab, and Norman cultures [21]. Similarly, in Central Crete, traditional methods such as boiling or pan-frying (and later, possibly, baking filled pies with pan-fried WVs) are prevalent in preparing wild greens, serving not only to enhance palatability but also to preserve nutritional integrity and slightly reduce natural bitterness [11]. These culinary practices are deeply rooted in historical contexts where wild greens were essential to everyday meals, providing communities with vital nutrients and flavours sourced directly from the natural environment [22,23]. Moreover, the cultural significance of wild greens extends beyond mere sustenance, encompassing rituals, festivals, and symbolic meanings embedded in local folklore and traditions [24]. Their culinary uses are, therefore, intimately intertwined with community identities and the preservation of cultural heritage, fostering a connection between people and their natural surroundings.

Moreover, the sensory classifications of wild greens—such as the aromatic, bitter, and non-bitter tastes most commonly perceived in the Mediterranean area—further elucidate the nuanced culinary preferences within Central Crete [11]. These sensory taxonomies guide culinary choices and reflect cultural attitudes towards specific plant compounds’ tastes and accompanying health benefits [7,9].

In this context, the unique characteristics of the Lasithi Plateau offer an especially compelling area of study within Crete, given its isolated environment and rich agricultural history. By narrowing our focus to Lasithi, this research aims to explore how the cultural and ecological specificities of this highland region may have influenced local practices in wild green foraging and consumption. This transition from general Mediterranean dietary elements to the unique biocultural environment of Lasithi enables a deeper understanding of the regional nuances and historical continuity embedded within the MD. Hence, our study aimed to contribute to the growing body of knowledge on wild food ethnobotany by investigating the consumption of wild greens (WGs) in the Lasithi area of Crete. The study’s specific aims were, therefore, (1) to document the local knowledge, practices, and perceptions surrounding the gathering and culinary use of WGs; (2) to evaluate possible relations between WVG tastes and their culinary preparations; (3) to compare the findings herein with those emerging from previously published food ethnobotanical studies conducted in the Eastern and Central Mediterranean, assessing possibly a specific Lasithian (“Minoan”) heritage. This study’s findings offer insights that extend beyond the Mediterranean context, providing a framework for understanding how sensory-driven preferences in traditional diets can guide the selection and preparation of wild food plants globally.

## 2. Materials and Methods

### 2.1. Study Area

**Figure 1:**
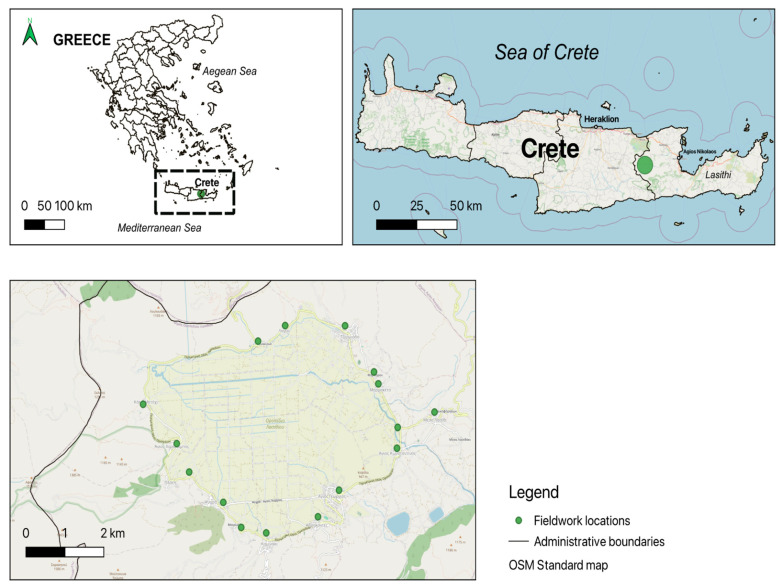
The maps show the location of Crete, the study area, and the villages explored in this study.

The study area of Lasithi (Figure 1), situated in eastern Crete, is renowned for its unique ecological and cultural characteristics. Historically shaped by fertile plains and a favourable mountainous climate encapsulated in a south Mediterranean island context, Lasithi has sustained diverse agricultural practices since ancient times. The study focuses on all the villages within the Lasithi Plateau; villages such as Tzermiado, known for its agrarian significance on the Lassithi Plateau, and Agios Georgios, with its traditional Cretan architecture and local farming practices, exemplify Lasithi’s rural heritage. Additionally, Kaminaki’s vineyards and production of local wines, along with Psichro’s association with the Diktean Cave and its archaeological significance, offer insights into the diverse facets of Lasithi’s cultural landscape [25]. These villages, including Plati, Agios Charalampos, Metochi, and Pinakiano, collectively showcase Lasithi’s heritage, which is nowadays mainly in the hands of elderly generations. Most villages are largely abandoned or depopulated since the middle and younger generations moved to the Cretan cities, elsewhere in Greece, or abroad. The study was conducted in the following settlements, which have nowadays between a few dozen and a few hundred permanent inhabitants: Tzermiado, Agios Georgios, Kaminaki, Psichro, Plati, Agios Charalampos, Metochi, Pinakiano, and Lagou.

### 2.2. Ecological and Historical Characteristics of the Study Site

The Cretan ecoregion exhibits a sharp altitudinal gradient. The low plains are warm and dry, with average annual temperatures between 17 and 19 °C and total rainfall of less than 300 mm in the southeastern parts of the island. In contrast, the higher elevations are cold and humid, with temperatures averaging 9–13 °C and annual rainfall reaching up to 1400 mm [11]. The island’s mountain ranges belong to the Alpine orogenic system, characterised by Mesozoic and Tertiary sedimentary rocks, with notable karstic landforms. Lasithi, located in eastern Crete, covers a diverse geographical area characterised by its fertile plains, rugged mountains, and coastal landscapes. The total surface area of Lasithi spans approximately 1818 square kilometres, making it one of the larger regional units on the island of Crete. The region’s altitude varies significantly, ranging from sea level along the northern coast to mountainous peaks over 2000 m above sea level in the Dikti Mountains. The Lassithi Plateau, a prominent geographical feature, sits at an average elevation of around 840 m and is a central agricultural hub within the region. The International Union for the Conservation of Nature (IUCN) recognises Crete as a “global centre of plant diversity and endemism”, being home and offering habitat to about 1800 species and subspecies of plants, of which 13% are locally endemic [26]. In Crete, anthropogenic activities have significantly impacted certain areas, primarily due to the overuse of pastures and grazing in mountainous regions, increasing tourism development, and some intensive agricultural practices in the southern coastal zone.

The Minoans flourished on the island of Crete by 2000 BC and developed advanced societies centred around palatial complexes such as Knossós, which became a hub of culture and trade in the Eastern Mediterranean. These centres were vital for distributing goods and resources, reflecting the Minoans’ sophisticated lifestyle and their possible reliance on wild plants as part of their diet [15]. Around 1500 BC, the influence of the Mycenaeans from mainland Greece led to significant cultural changes on the island, which may have affected local food traditions, including the foraging and use of wild greens [11]. The Lasithi region, believed to have served as a refuge for the Minoans during this period, presents a unique setting where these ancient practices could have been preserved, offering a glimpse into the enduring impact of Minoan ecological adaptations on the culinary heritage of Crete [11].

### 2.3. Data Collection

Ethnobotanical field research was conducted in March 2024 across several villages in Lasithi, Crete, as depicted in Figure 1. The area has maintained its traditional agro-pastoral activities up to the present day, even if practised only by a few remaining elderly inhabitants. Tourist development in these areas remains light and does not follow the rapid growth rate observed in other parts of Crete. Locals are mainly elderly farmers and shepherds; at the moment, primarily elderly community members live in the area all year around.

In total, 31 participants were selected using purposive sampling and the snowball technique [27], focusing on individuals aged 40 to 84 years, mainly including farmers, shepherds, and elderly women known for their extensive knowledge and practices linked to nature and local flora. Participants were approached in streets, village squares, and local markets. Verbal informed consent was obtained from all participants before conducting semi-structured interviews, adhering to the ethical guidelines set forth by the International Society of Ethnobiology [28]. Semi-structured interviews were conducted in either modern Greek or English. For each of the WGs free listed during the study, participants were asked to provide the local name, part used, local food use, and mode of preparation. The survey excluded edible mushrooms and wild fruits.

The quoted wild food taxa were collected and identified on-site using standard reference works concerning the Aegean flora [29]; identifications were later cross-checked with Cretan annotated checklists [30,31]. Voucher specimens (coded UVVETBOTCr01-54) were deposited at the Herbarium of the Bio-Cultural Diversity Lab of the Department of Environmental Sciences, Informatics, and Statistics, Ca’ Foscari University of Venice, Italy. For taxa that could not be identified in the field, their identification relied on detailed descriptions of plant morphology, ecology and sensory characteristics provided by participants. Photographs of presumed plants were also shown to the participants for verification after initial evaluation of local names and descriptions. Nomenclature followed the World Flora Online database, and plant family names were consistent with the Angiosperm Phylogeny Website [32]. Local Greek names were transliterated into the Latin alphabet directly from phonetic transcriptions of original recordings.

### 2.4. Data Analysis

The findings were qualitatively compared with data on wild greens and their local uses previously collected by our or other colleagues’ research groups in recent years in other Eastern and Central Mediterranean regions (for methods and compared areas, see [9]). Moreover, we also quantitatively and explicitly compared the Lasithi data with other data from Central Crete, Sicily, and Karpathos. Our focus excluded broader national or regional reviews lacking transparent descriptions of the field methodologies or modern ethnobiological methods, such as those without first-hand interviews with locals or comprehensive documentation of local plant names.

For Lasithi and the other comparative study sites where we conducted fieldwork (Central Crete and Karpathos), we employed emic and etic approaches to sensory classification. In Lasithi, the emic classification was based on interviews with local respondents, capturing their spontaneous descriptions of WGs. Conversely, the etic classification related to the Sicilian study was presumed from the botanical identity across all locally studied regions. This methodological approach allowed us to comprehensively analyse and compare how sensory perceptions, local culinary uses, plant part used, and quotation cultural uses of wild greens may vary across Lasithi, Central Crete, Karpathos, and Sicily, contributing to a nuanced cross-cultural understanding of these traditional food practices. We constructed a matrix where the most frequently mentioned wild green (WG) genera in each region were listed in rows, and columns represented local culinary uses, part used, and sensory classifications. The sensory classifications included the ‘bitter/pungent’, ‘non-bitter’, or ‘aromatic’ categories (‘b’, ‘nb’, and ‘ar’, respectively).

The data analysis used SAS 9.4 software (SAS Institute Inc., Cary, NC, USA) to explore relationships among variables within the study regions. Descriptive statistics utilised PROC FREQ to analyse the distribution of categorical variables: site (LAS = Lasithi; CCRE = Central Crete; KAR = Karpathos; SIC = Sicily), sensory classification (Aromatic, Non-Bitter, Bitter), quotation uses, and local culinary uses.

Correspondence analysis, employing PROC CORRESP, was used to examine the relationships between categorical variables, to identify associations between WGs and culinary methods, and to highlight common preparation practices. Finally, logistic regression analysis using odds estimates was employed to model the effects of various independent variables on dependent variables. Logistic regression analysis helped assess how taste attributes, particularly bitterness, influenced WG selection and preparation choices. An integrated model was developed with the following formulation:

logit(P(Y = 1)) = log(P(Y = 1)/(1−P(Y = 1))) = β0 + β1X1 + β2X2 + ⋯ + βkXk

where

P(Y = 1) is the probability of the outcome being 1 (using wild greens for boiling).
logit(P) is the log-odds of the probability.
β0 is the intercept of the model.
β1, 2, …, βk are the coefficients for the predictor variables.
X1, 2, …, Xk are the predictor variables (region, local sensory classification).

Site categories were qualitatively defined as CCRE (0), KAR (1), SIC (2), and LAS (3). Sensory classifications were defined as aromatic (0), non-bitter (1), and bitter (2).

The significance of each coefficient (*p* < 0.05) was assessed to determine the influence of site, local sensory classification, and their interaction on outcomes. Model performance was validated using the Hosmer–Lemeshow test for goodness-of-fit and the area under the ROC curve (AUC) for predictive accuracy. This approach facilitated a comprehensive understanding of the data, revealing nuanced relationships between categorical variables and outcomes of interest.

## 3. Results

### 3.1. Diversity of WGs Foraged and Consumed in Lasithi

Table 1 reports the WGs documented in the field study through interviews with study participants and notes the use in the study site of 59 folk taxa corresponding to 58 botanical taxa of 21 botanical families (2 were only identified at the genus level and another was identified at the family level. Asteraceae was the most represented family, with 18 species, including some frequently cited ones like *Cichorium intybus* and *Sonchus oleraceus*. Other significant families included Apiaceae, with examples like *Foeniculum vulgare*; Amaranthaceae, including *Amaranthus blitum*; and Lamiaceae with *Origanum vulgare* subsp. *hirtum* (Link) A.Terracc. These plants are commonly found in uncultivated lands, field margins, and dry meadows, with some growing in woods and rocky areas. The plants we reported were primarily harvested for their aerial parts—leaves, tender shoots, and basal rosettes—though some, like *Muscari comosum*, were also mentioned for their edible bulbs. These parts we reported as used in traditional dishes, especially as boiled vegetable mixes, *chortopita* (savoury pies made with dairy products, and wild greens and cultivated beet, *Beta vulgaris*, filling), and salads, with most of the plants consumed in cooked forms. The frequency of use varied, with 13 species (including *Taraxacum hellenicum*) cited by many respondents. Many species we reported as commonly gathered and consumed, while others, like *Rumex crispus*, were less frequently mentioned. None of our study participants reported toxicity issues of the documented species; however, some studies highlighted the presence of toxic compounds in some species such as *Echium italicum* [33].

### 3.2. Comparative Analysis of Lasithi’s WGs with Those of Other Mediterranean Areas

#### 3.2.1. Lasithi’s WGs in the Eastern and Central Mediterranean Spectrum

**Figure 2:**
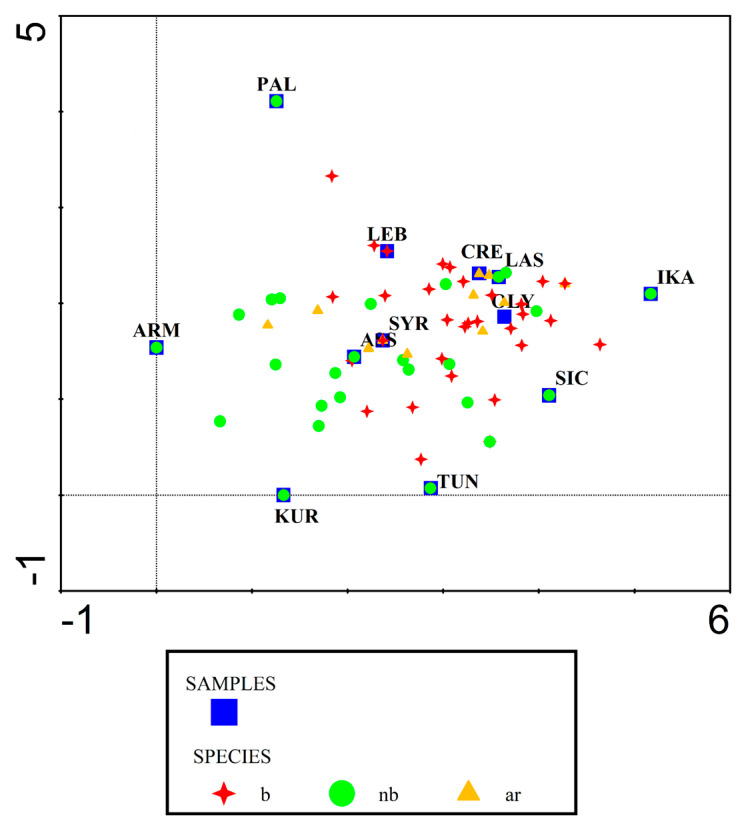
DCA analysis showing Lasithi’s WG foraging (ar: aromatic; b: bitter; nb: non-bitter) compared with that of other Mediterranean areas (LAS: the present study; CRE: Central Crete [11]; OLY: Olympos, Northern Karpathos Island, Greece [34]; LEB: Lebanon [35]; ASS: Assyria [36]; SYR: Coastal Syria [19]; SIC: Sicily [20]; IKA: Ikaria Island, Greece [9]; PAL: Palestine [37]; ARM Armenia [38]; KUR Kurdistan [36]; TUN: Tunisia [39].

Figure 2 shows that Lasithi’s WGs’ foraging patterns are similar to those of Central Crete and Northern Karpathos (Olympos) and, to a lesser extent, to Lebanese foraging. This can result from a similar ecology and biocultural/ethnobotanical heritage.

#### 3.2.2. Patterns of WG Use in Lasithi Compared with Those of Central Crete, Karpathos, and Sicily

The use of wild greens (WGs) across Mediterranean regions reveals distinct regional preferences in sensory attributes, preparation methods, and plant parts utilised (Table 2). In Lasithi (LAS), where 59 WGs (folk taxa) were documented, bitterness is the predominant sensory attribute, characterising 45.76% of the WGs. Moreover, the differentiation of *Crepis* spp. into three folk linguistic categories in Lasithi (Table 1), suggests a sophisticated perception of distinguishing different forms, shapes, and tastes of a prominently bitter botanical genus by the local population. This could be attributed, of course, not only to their morphological appearance (which is somewhat similar to unskilled eyes) but also to a sophisticated perception of the plants’ bitterness, which may drive their diverse specific uses in traditional dishes as valued by the local community. The most common preparation method reported was boiling, used for 83.05% of the WGs. Also, in Central Crete (CRE), bitter WGs (botanical species) are the most common, comprising 50% of the total. Boiling is the primary preparation method for 66% of the WGs, suggesting locals’ favoured cooking method. Also, in Karpathos (KAR), with 78 documented WGs, there is a preference for bitter WGs, making up 35.59% of the total. The non-bitter WGs accounted for 27 out of 78 (34.61%) of the WGs, while aromatic WGs comprised 16 out of 78 (20.51%) of the WGs. Raw consumption was notable here, accounting for 46.15% of the WGs, highlighting a distinct preference for freshly consumed greens. Sicily (SIC), where the previous survey we considered in this comparison examined 253 WGs, has revealed a balanced sensory profile with a nearly equal representation of non-bitter WGs (41.93%) and bitter WGs (49.4%). Boiling is a less prevalent preparation method in Sicily, and it is used for only 8.30% of WGs, while other cooking methods are predominant (51.78%).

Overall, while boiling was more emphasised in Lasithi, Central Crete highlights a balance between bitter and non-bitter varieties with a strong focus on leaves. Karpathos shows a strong preference for raw consumption and leaves, and Sicily presents a balanced sensory profile with a wide range of culinary applications, including significant use of cooking. These regional differences underscore the rich diversity in Mediterranean wild vegetable usage and provide insights into these regions’ distinct culinary traditions and practices.

#### 3.2.3. Regional Variations in WGs’ Culinary Transformations and Consumption

**Figure 3:**
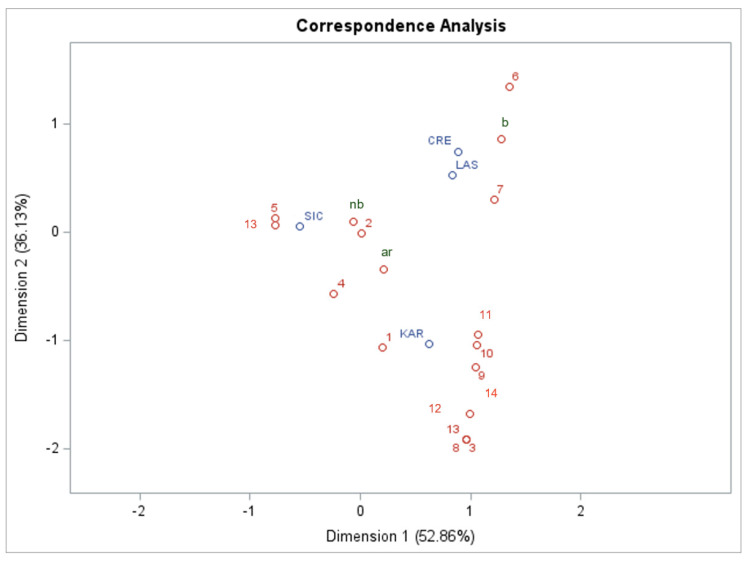
Greek regional differences in WVG culinary practices were revealed by correspondence analysis. Summary of sensory classification (b: bitter; nb: non-bitter; ar: aromatic) and local food use typologies: 1. consumed raw; 2. boiled; 3. fried and pie filling; 4. roasted; 5. cooked in omelettes; 6. cooked in mixtures; 7. salads; 8. with legumes; 9. pickled; 10. seasoning; 11. miscellaneous; 12. boiled and after pan-fried; 13. consumed raw or cooked (other than boiling); 14. consumed raw or boiled.

The correspondence analysis (CA) of the wild green (WG) data across Central Crete, Karpathos, Lasithi, and Sicily (Figure 3) revealed notable regional differences and similarities in sensory classifications and culinary practices. The analysis indicated that the first two dimensions captured most of the variance in the dataset, with Dimension 1 explaining 53% and Dimension 2 36%. Central Crete was prominently positioned along Dimension 1, reflecting its distinctive sensory and culinary practices. In contrast, Karpathos showed a mixed profile with a strong negative association with Dimension 2, suggesting a preference for specific sensory attributes, such as bitterness, that differentiate it from other regions. Lasithi exhibited positive associations in both dimensions, indicating a blend of regional practices and shared characteristics with Central Crete and Karpathos. Sicily stood out with a unique profile for Dimension 1, indicating a distinct approach to sensory and culinary uses of WGs. These associations can highlight the rich diversity of using and classifying WGs across the Mediterranean and the specific regional practices in different regions, thus contributing to a broader ethnobotanical understanding. Lasithi’s convergence with Central Crete and Karpathos underscored its role as a meeting point for diverse traditional practices, while Sicily’s distinct profile emphasised the influence of local culinary traditions.

#### 3.2.4. Greek Regional Differences in WG Preparation

The analysis showed that Lasithi (LAS) had a markedly higher likelihood of boiling WGs than Central Crete (CRE). The odds ratio (OR) for Lasithi is 2.201 (95% CI: 1.095–4.424, *p* < 0.0001), indicating that WGs in Lasithi were more than twice as likely to be boiled than in Central Crete. This trend suggests a strong cultural preference in Lasithi for boiling as a primary preparation method, possibly tied to traditional culinary practices that emphasise transforming raw ingredients through cooking.

In contrast, Karpathos (KAR) exhibited a significantly lower likelihood of using WGs for boiling, with an OR of 0.586 (95% CI: 0.307–1.119, *p* = 0.013). This lower probability suggests that other cooking methods, such as raw consumption or different forms of cooking, are more prevalent on the island. Sicily (SIC) also showed a reduced probability of boiling WGs compared to Central Crete, with an OR of 0.561 (95% CI: 0.323–0.975, *p* = 0.0002). The findings from Karpathos and Sicily can highlight regional variations where boiling is less dominant, pointing to diverse culinary traditions that value different preparation techniques.

#### 3.2.5. Sensory Classification and Its Impact on WG Preparation

The sensory classification of WGs plays a crucial role in determining their preparation methods. The analysis revealed that aromatic WGs were significantly more likely to be boiled than non-bitter WGs. The OR for aromatic WGs was 1.659 (95% CI: 1.246–2.209, *p* < 0.0001), indicating a strong preference for boiling aromatic plants (Table 3). This could be due to how boiling helps release and enhance the aromatic compounds, making these WGs more flavourful and suitable for traditional dishes where their aroma is crucial.

On the other hand, bitter WGs were less likely to be boiled than non-bitter ones, with an OR of 0.353 (95% CI: 0.245–0.511, *p* < 0.0001). This trend suggests that bitterness, a sensory attribute often perceived as less desirable, may lead to alternative preparation methods that mask or reduce the bitter taste. For instance, bitter greens might be more commonly used in raw preparations when combined with other ingredients to balance their flavour, or cooked in ways that minimise their bitterness.

## 4. Discussion

The current study reveals a significant diversity in the botanical families and species of wild greens (WGs) utilised across the Mediterranean, underscoring the rich ethnobotanical heritage of the region. Among the herein-studied areas, Sicily emerges as a biodiversity hotspot, hosting 39 botanical families and 253 species. This richness reflects Sicily’s varied ecological niches, shaped by its complex geomorphology and historical influences. The island’s diverse landscapes, including coastal areas, plains, and mountainous regions, support many foraged wild-growing plant species. Additionally, Sicily’s historical role as a cultural crossroads, influenced by civilisations such as the Greeks, Romans, and Arabs, has enriched its botanical heritage by introducing and cultivating numerous plant species [21,40]. These factors have likely contributed to Sicily’s unique food ethnobotanical diversity, which has been further supported by the fact that the study we used for comparison results from extensive data collection across diverse landscapes.

In contrast, Lasithi, herein studied for the first time, with 22 families and 59 species (LAS), and Central Crete (CCRE), where 24 families and 54 WGs have been recorded, present different biocultural diversity patterns. The lower diversity in Lasithi and CCRE can be attributed to the study area’s more restricted spatial and ecological extent, i.e., foraging practices focused on a narrower range of ecozones and geographical sites [41]. Despite this, the WGs used in Lasithi highlight some critical characteristics. This study exhibited a relatively high percentage of bitter-tasting plants (45.76%), suggesting that the local culinary tradition naturally preferred bitter plant foods. It would be fascinating to intersect this finding with historical, archaeobotanical, and archaeological data regarding the ancient Minoans to assess whether bitter foods were prevalent among them. To date, the bitter plants in Lasithi are often cited by locals as suitable for their health-promoting properties, aligning with traditional Mediterranean dietary practices that emphasise the importance of consuming bitter plants and with contemporary nutraceutical studies that underline the crucial role of consuming bioactive-rich foods [11,15,42,43,44].

The Central Crete study (CCRE), with its 24 families and 54 species, showed similarities to the Lasithi study regarding diversity but differed somewhat in its culinary practices and species selection. While CCRE shared some species with Lasithi, it also incorporated other WGs that reflected the site’s specific differences in microclimates and historical agricultural practices [11].

The Northern Karpathos study (KAR) presents another distinct biodiversity pattern with 30 botanical families and 78 documented WGs. Compared to CCRE and LAS, KAR’s relatively higher WG diversity reflects the island’s long isolation and, possibly, a more conservative LEK and food heritage, which is fading at a comparatively lower pace. KAR’s unique ecological zones and historical legacy, linked to its Doric heritage and long isolation, may have contributed to a rich WG use tradition marked by raw snacks, especially less aromatic WGs and their cooked preparations [34,45].

The culinary traditions related to WGs in LAS, KAR, CCRE, and SIC (Sicily) reflect a rich tapestry of cultural, practical, and historical influences. In Lasithi, boiling was reported as a prevalent method (54.55%) used to prepare WGs, particularly those with bitter flavours. This method likely mitigates bitterness and enhances the digestibility of WGs by breaking down tough fibres and reducing potentially harmful compounds [46]. Boiling as a culinary practice developed during the Neolithic Revolution and has probably been incredibly efficient in rural settings with limited resources, ensuring sustenance and nutritional value from wild plant sources. Research findings support this argument as boiling is able to reduce bitterness at least to some extent and can preserve essential nutrients, thus highlighting how such cultural practices cater to local tastes and may optimise the health benefits derived from WGs [45,47,48,49]. Similarly, data previously collected in Central Crete demonstrate a clear preference for boiling (50%) as the primary method of WG preparation. The focus on boiling in Central Crete, similar to Lasithi, may be attributed to the need to slightly reduce bitterness and improve the palatability of certain WGs, which aligns with the broader Cretan dietary heritage focusing on health and nutrition. This practice also underscores the adaptability of traditional culinary techniques to the specific plant species and flavours available in the local natural environment [50,51].

In contrast, Karpathos (KAR), reporting 78 documented WGs, displays a distinct preference for the raw consumption of WGs (61.82%), which may have been influenced by the island’s Doric cultural legacy, as argued in previous studies [50]. Consuming freshly foraged plants immediately after harvesting preserves their natural flavours and nutritional integrity, aligning with modern dietary trends that emphasise the health benefits of consuming vegetables in their uncooked state. Such research lines indicate that raw vegetable consumption retains more vitamins, minerals, and enzymes than cooked vegetables, contributing to higher dietary fibre intake and antioxidant levels [44,52,53,54,55]. These findings reinforce the cultural wisdom behind Northern Karpathos’ matrifocal, rich folklore [45].

Sicily, on the other hand, exhibits a clearly balanced preference for both bitter and non-bitter WGs, with a significant emphasis on cooking methods such as boiling (63.75%) [20]. Sicilian cuisine is known for its rich culinary heritage and multi-layered cultural fusions, where cooking techniques like frying, baking, and stewing enhance the flavours of WGs. These culinary practices are also reflected by the contemporary innovations generated by chefs and home cooks and their sophisticated dishes tailored to seasonal availability and festive traditions [56].

The regional variations in the use and preparation of WGs across Lasithi, Karpathos, Central Crete, and Sicily underscore the intricate interplay of historical continuity, ecological adaptation, and cultural significance. These culinary practices highlight how local environments shape food traditions, from mitigating bitterness through boiling in Lasithi and celebrating natural flavours in raw consumption on Karpathos to creating elaborate dishes in Sicily that honour a diverse culinary heritage. These insights into regional foodways enrich our understanding of Mediterranean diets and highlight the resilience and innovation embedded in culinary traditions across time and space.

The quotation index that measures the frequency of references to specific WG species provided valuable insights into the cultural and culinary landscape of Lasithi within the context of the Mediterranean region. In Lasithi, 47.27% of the species were commonly quoted and such a high proportion may underscore the enduring cultural significance and widespread utilisation of these plants in local cuisines and traditional medicinal practices [49]. The consistent references to certain species reflect a deep-rooted connection between the community and these WGs, indicating their integral role in daily culinary traditions and healthcare practices that have been shaped over generations [57].

The significance of bitterness in traditional diets extends beyond mere taste preferences, as it often carries deep cultural meanings and health implications. Across various Mediterranean regions, including Lasithi, bitter greens are celebrated not only for their distinctive flavours but also for their associated health benefits. However, Sansanelli et al. [58] report in a study in South Italy that preference/degree of bitterness acceptance varies depending on individual perception. Beyond Mediterranean, Singh et al. [59] report that participants in the district of Kashmir Himalaya in India replace the first boiled water with fresh water when boiling bitter-tasting plants, which reflects the local non-acceptance of bitter taste.

Studies in ethnobotany and culinary anthropology emphasise the importance of high quotation indexes, suggesting that commonly quoted WGs are not merely ingredients but also symbols of cultural identity and repositories of traditional knowledge [11]. In Lasithi, these species likely represent a blend of historical continuity and practical applications handed over through oral traditions and integrated into local diets and medicinal practices. This trend may highlight the critical role that WGs play in preserving the cultural heritage of Lasithi and maintaining the region’s biodiversity and culinary diversity.

### 4.1. Implications of the Findings

The findings reported herein underline the significant role that both regional culinary traditions and the sensory attributes of plants play in the preparation methods of wild greens. The marked differences between regions like Lasithi, where boiling is dominant, and Karpathos or Sicily, where it is less common, reflect deep-rooted cultural practices and preferences. Furthermore, the sensory characteristics of WGs, particularly bitter and aromatic tastes, are crucial determinants of how these plants are utilised in local cuisines. This interplay between taste and tradition highlights the diversity of Mediterranean culinary practices and offers insights into how regional identities are expressed through food.

By understanding these sensory influences, we better appreciate the complex decisions behind cooking wild greens, revealing how taste and culture intertwine to shape local culinary landscapes.

### 4.2. Implications for Further Research

Our study opens several avenues for future research to understand the intricate dynamics of culinary practices, ethnoecological adaptations, and cultural preferences surrounding wild greens in Mediterranean regions. One promising area of investigation is the role of taste perception genetics in influencing dietary choices and expected health outcomes. Understanding how genetic predispositions, such as sensitivity to bitterness (e.g., PROP sensitivity and beyond), shape individual and collective preferences for wild greens could provide deeper insights into why certain plants are favoured or avoided in specific culinary traditions.

Recent advances in molecular biology and genetics offer tools to explore how genetic diversity within populations contributes to variations in taste perception and food preferences [60,61]. By integrating genetic analyses with ethnobotanical studies, researchers can elucidate how ancient genetic adaptations to local environments continue to influence contemporary dietary habits, thereby connecting cultural practices with biological mechanisms.

Moreover, our findings suggest the importance of investigating socio-economic factors that influence the utilisation of wild greens. Exploring how urbanisation, globalisation, and economic development impact traditional food systems can reveal patterns of change in culinary diversity and dietary habits over time. Socio-economic studies in similar contexts have shown how market integration, changing lifestyles, and access to alternative foods influence the consumption of wild greens and traditional foods [19,62,63].

Furthermore, there is a critical need to examine strategies for promoting biodiversity conservation and sustainable food systems in Mediterranean regions and globally. Wild greens play a crucial role in local biodiversity and ecosystem health, yet their use faces challenges due to habitat loss, overharvesting, and climate change. Future research could focus on identifying culturally appropriate conservation strategies that integrate local knowledge systems with conservation science, foster sustainable harvesting practices, and enhance the resilience of wild vegetable populations.

## 5. Conclusions

In conclusion, our study reveals the particular uses of wild greens in the Lasithi Plateau, eastern Crete, the presumed last refugium of the Minoan civilisation. We observed a remarkable diversity of WGs and a considerable, nuanced local knowledge of bitter greens. Compared with other Greek or Greek-influenced areas, boiling bitter wild plants in Lasithi seems prevalent. Our findings suggest that the Minoan diet could have been particularly bitter and, more generally, highlight how taste perception shapes local culinary traditions. Our study highlights that the significance of bitterness in traditional diets extends beyond taste preferences, as it often carries deep cultural meanings and health implications. These insights deepen our understanding of how Mediterranean societies have historically interacted with their natural environments and articulated their food culture, showcasing the potential of wild greens to contribute to sustainable diets and global health. Ongoing historical, botanical, and pharmacological research and bio-cultural conservation efforts are essential to preserve and promote these valuable wild plant resources’ cultural heritage and ecological diversity. Future research could consider the genetic basis of taste preferences, especially bitterness sensitivity, to investigate how these influence culinary traditions and health outcomes in Mediterranean diets.

## Figures and Tables

**Table 1:** Wild greens and their local food uses as reported at the study site (the table also includes a few cultivated species whose food use is particular).

Botanical Name	Family	Voucher Code	Local Names	Part Used	Local Food Use	Quotation Index
*Allium ampeloprasum* L.	Amaryllidaceae	Cr01	Agriòpraso	Whole plant	Seasoning, chortopita	***
*Amaranthus blitum* L.	Amaranthaceae		Vlito	Leaves	Boiled mixes, cooked with potatoes and taro	***
*Asparagus aphyllus* L.	Asparagaceae	Cr33	Agriasfaradzia	Shoots	Omelettes	***
*Asphodeline lutea* Rchb.	Asphodelaceae	Cr06	Drili, Drilos, Grilos	Young aerial parts	Boiled mixes	**
*Beta vulgaris* L. CU	Amaranthaceae	Cr03	Ghula, Seskula	Leaves	Boiled mixes, chortopita	**
*Centaurea calcitrapa* L.	Asteraceae	Cr39	Adriana, Andriana	Rosettes	Boiled mixes	**
*Centaurea raphanina* subsp. *raphanina*	Asteraceae	Cr46	Charachuli	Rosettes	Boiled mixes	**
*Chondrilla juncea* L.	Asteraceae		Ampeloradiko, Prikosirida, Radiči	Rosettes	Boiled mixes	**
*Cichorium intybus* L.	Asteraceae		Agrioradiki, Radiči	Rosettes	Boiled mixes	**
*Cichorium spinosum* L.	Asteraceae	Cr32	Stamnagathi	Rosettes	Salads, boiled mixes	***
*Crepis* sp.	Asteraceae		Bodičina, Boudičinači	Rosettes	Boiled mixes, salads	**
*Crepis sancta* (L.) Bornm.	Asteraceae	Cr45	Agrioradiko, Chorto, Papafkači, Papaftači, Prikosirida, Prikovruves, Radiči, Staridha	Rosettes	Boiled mixes	***
*Crepis vesicaria* L.	Asteraceae	Cr10, Cr28	Chočinogouli, Koučinogouli	Rosettes	Boiled mixes	**
*Cynara cornigera* Lindl.	Asteraceae	Cr08	Agriadzinara	Stems, flower receptacles	Cooked, omelettes	**
*Daucus carota* L.	Apiaceae	Cr11	Stafilìnakas	Young aerial parts	Boiled mixes, chortopita	***
*Dioscorea communis* (L.) Caddick & Wilkin	Dioscoreaceae		Àvronies	Young shoots	Omelettes, boiled mixes	**
*Echium italicum* L.	Boraginaceae		Chorto, Pederamia	Rosettes	Boiled mixes	*
*Erodium cicutarium* (L.) L’Hér.	Geraniaceae	Cr22	Persika	Leaves	Boiled mixes	*
*Eryngium creticum* Lam.	Asteraceae		Agriagathi	Rosettes	Boiled mixes	*
*Foeniculum vulgare* Mill.	Apiaceae	Cr09	Maratho	Young aerial parts	Boiled mixes, chortopita, seasoning	***
*Glebionis coronaria* (L.) Spach	Asteraceae	Cr05	Madilida	Young leaves	Boiled mixes, chortopita	**
*Helminthotheca echioides* (L.) Holub	Asteraceae		Shiromurida	Rosettes	Boiled mixes	***
*Hypochaeris glabra* L.	Asteraceae		Archondovrouva, Archodouvouva, Kafkalidha, Mirtiso, Xilochorto	Rosettes	Boiled mixes	**
*Lactuca serriola* L.	Asteraceae	Cr49	Agriomàrulo	Rosettes	Boiled mixes	**
*Lactuca virosa* L.	Asteraceae		Chorto	Rosettes	Boiled mixes	*
*Leontodon* sp.	Asteraceae		Prikorodiko	Rosettes	Boiled mixes	*
*Leontodon tuberosus* L.	Asteraceae	Cr35	Pendanevro, Visorodika	Whole young plant	Boiled mixes	**
*Muscari comosum* (L.) Mill.	Asparagaceae		Askordoulakos	Bulbs	Boiled, salads	**
*Notobasis syriaca* (L.) Cass.	Asteraceae	Cr50	Agavanos	Rosettes	Boiled mixes	*
*Onopordum tauricum* Willd.	Asteraceae		Koufoutì	Flower receptacles	Cooked	**
*Origanum vulgare* subsp. *hirtum* (Link) A.Terracc.	Lamiaceae		Rigani	Flowering tops	Seasoning	*
*Papaver rhoeas* L.	Papaveraceae	Cr20	Agoutzounada, Koutzounada	Rosettes	Boiled mixes, chortopita	***
*Petromarula pinnata* (L.) A. DC.	Campanulaceae	Cr23	Maroullida	Leaves	Salads, boiled mixes	**
*Portulaca oleracea* L.	Portulacaceae		Glistrida	Aerial parts	Salads	*
*Prasium majus* L.	Lamiaceae	Cr17	Lagoudochorto	Leaves	Boiled mixes, salads	*
*Ranunculus ficaria* L.	Ranunculaceae		Fasolida	Rosettes	Boiled mixes	*
*Raphanus raphanistrum* L.	Brassicaceae		Rapagnida	Young aerial parts	Boiled mixes	***
*Reichardia picroides* (L.) Roth	Asteraceae	Cr18	Galatzida, Galatzina	Rosettes	Salads, boiled mixes	**
*Rumex crispus* L., *R. obtusifolium* L. and possibly other *Rumex* spp.	Polygonaceae		Labatho, Lapatho	Leaves	Dolmades, boiled mixes, fried	**
*Salvia fruticosa* Mill.	Lamiaceae		Kinomalìa	Galls	Snack	*
*Scabiosa columbaria* L.	Caprifoliaceae	Cr47	Stavrochorto, Stavroxilo, Stravoxilo	Rosettes	Boiled mixes	***
*Scandix pecten-veneris* L.	Apiaceae	Cr12	Achaziči, Archadikos, Mirisača, Mirisači	Young aerial parts	Boiled mixes, chortopita	**
*Scolymus hispanicus* L.	Asteraceae	Cr25	Askolimbi, Askolimbros, Askorduolakas	Young aerial parts and upper outer root parts after removing thorny portions	Boiled, omelettes	***
*Silene vulgaris* (Moench) Garcke	Caryophyllaceae		Strufulia	Young aerial parts	Boiled mixes	**
*Sinapis alba* L.	Brassicaceae	Cr16	Lapsanides, Nerovruves, Vrouves	Young aerial parts	Boiled mixes	***
*Sinapis arvensis* L.	Brassicaceae		Lapsanides, Nerovruves, Vrouves	Young aerial parts	Boiled mixes	***
*Solanum nigrum* L.	Solanaceae		Stroufigas, Stroufika	Leaves	Boiled mixes, cooked with potatoes and taro	***
*Sonchus asper* (L.) Vill.	Asteraceae		Tzochos	Rosettes	Boiled mixes	***
*Sonchus oleraceus* L.	Asteraceae	Cr26	Tzochos	Rosettes	Boiled mixes	***
*Taraxacum hellenicum* Dahlst.	Asteraceae	Cr40	Kalitza, Karnovisa, Kofta, Kofti	Leaves	Salads, boiled mixes	**
*Tordylium apulum* L.	Apiaceae	Cr02	Kafkalidha	Young aerial parts	Boiled mixes, chortopita	**
*Tragopogon porrifolius* L.	Asteraceae		Agouduras, Skoulos	Young shoots	Salads, boiled mixes	**
*Urtica* spp.	Urticaceae	Cr52	Tzouknida	Leaves	Boiled mixes, chortopita	**
*Vicia faba* L. CU	Fabaceae	Cr54	Koučan	Shoots	Salads	*
*Vitis vinifera* L. CU	Vitaceae		Ampeli	Shoots	Boiled mixes, dolmades	*
*Urospermum picroides* (L.) Scop. ex F.W.Schmidt	Asteraceae	Cr43	Chodrolachanida, Lachanida	Rosettes	Boiled mixes	**
Unidentified taxa	Fabaceae		Levan	Young fruits	Snack	*

CU: cultivated taxa; chortopita: typical Greek pie filled with (wild) greens and sometimes cheese; *** quoted by more than 40% of the study participants; ** quoted by 10–39% of the study participants; * quoted only by 1, 2, or 3 study participants.

**Table 2:** Comparison of local food uses of the WG taxa in Lasithi and the three Greek or Greek-influenced regions (Central Crete, Karpathos, and Sicily, respectively); * data from KAR and SIC included a few raw wild plant snacks (*n* = 12 in KAR and a non-specified number in SIC), while consumption of these was not considered in the field studies conducted in LAS and CRE.

Category	LAS (*n* = 59)	CRE (*n* = 54)	KAR (*n* = 78)	SIC (*n* = 253)
Sensory Classification *				
Aromatic (ar)	6 (10.16%)	5 (9.26%)	16 (20.51%)	22 (8.69%)
Bitter (b)	27 (45.76%)	27 (50.00%)	28 (35.59%)	125 (49.40%)
Non-Bitter (nb)	26 (44.08%)	18 (33.33%)	27 (34.61%)	106 (41.93%)
Local Culinary Uses *				
Raw	2 (3.4%)	6 (12.00%)	36 (46.15%)	95 * (37.55%)
Boiled	49 (83.05%)	33 (66.00%)	21 (26.92%)	21 (8.30%)
Cooked (in other ways)	7 (11.86%)	10 (20.00%)	15 (19.23%)	131 (51.78%)
Seasoning	1 (1.69%)	1 (2.00%)	6 (7.69%)	6 (2.37%)
Part Used *				
Leaves	13 (22.03%)	22 (40.00%)	40 (51.28%)	80 (31.63%)
Young Aerial Parts	10 (16.94%)	18 (33.33%)	1 (1.28%)	64 (25.30%)
Flowers/Inflorescences	2 (3.38%)	2 (3.70%)	4 (5.13%)	20 (7.91%)
Bulbs/Tubers	1 (1.69%)	1 (1.85%)	3 (3.85%)	13 (5.15%)
Aerial Parts	2 (3.38%)	1 (1.85%)	14 (17.95%)	0 (0.00%)
Roots/Stems	0 (0.00%)	0 (0.00%)	0 (0.00%)	6 (2.37%)
Fruits	1 (1.69%)	0 (0.00%)	14 (17.95%)	1 (0.40%)
Whole Plant	1 (1.69%)	1 (1.85%)	0 (0.00%)	0 (0.00%)
Miscellaneous	29 (49.20%)	9 (16.67%)	2 (2.56%)	69 (27.27%)

* Sensory classification (LAS = 59; CRE = 50; KAR = 71; SIC = 253); local culinary uses (LAS = 59; CRE = 50; KAR = 78; SIC = 253); part used (LAS = 59; CRE = 54; KAR = 71; SIC = 253).

**Table 3:** Logistic regression analysis of factors influencing the boiling preparation of WGs across Greek regions and Sicily.

Category	Effect	Estimate (β)	Standard Error	*p*-Value	Odds Ratio (OR)	95% Confidence Limits
**Site**	Region (Ref. CRE)					
	Region KAR	−0.454	0.182	0.013	0.586	0.307 to 1.119
	Region LAS	0.870	0.207	<0.0001	2.201	1.095 to 4.424
	Region SIC	−0.497	0.135	0.000	0.561	0.323 to 0.975
**Local Sensory Classification**	(Ref. Non-Bitter)					
	Aromatic (ar) vs. Ref	0.506	0.130	<0.0001	1.659	1.246 to 2.209
	Bitter (b) vs. Ref	−1.043	0.192	<0.0001	0.353	0.245 to 0.511

## Data Availability

The original contributions presented in the study are included in the article, further inquiries can be directed to the corresponding authors.

## References

[1] Tiffon C. (2018). The impact of nutrition and environmental epigenetics on human health and disease. Int. J. Mol. Sci..

[2] Ordovas J.M., Ferguson L.R., Tai E.S., Mathers J.C. (2018). Personalised nutrition and health. BMJ.

[3] Serra-Majem L., Bach-Faig A., Raidó-Quintana B. (2012). Nutritional and cultural aspects of the Mediterranean diet. Int. J. Vitam. Nutr. Res..

[4] Guasch-Ferré M., Willett W.C. (2021). The Mediterranean diet and health: A comprehensive overview. J. Intern. Med..

[5] Obeid C.A., Gubbels J.S., Jaalouk D., Kremers S.P.J., Oenema A. (2022). Adherence to the Mediterranean diet among adults in Mediterranean countries: A systematic literature review. Eur. J. Nutr..

[6] Des Gachons C.P., Uchida K., Bryant B., Shima A., Sperry J.B., Dankulich-Nagrudny L., Tominaga M., Smith A.B., Beauchamp G.K., Breslin P.A. (2011). Unusual pungency from extra-virgin olive oil is attributable to restricted spatial expression of the receptor of oleocanthal. J. Neurosci..

[7] Cui M., Chen B., Xu K., Rigakou A., Diamantakos P., Melliou E., Logothetis D.E., Magiatis P. (2021). Activation of specific bitter taste receptors by olive oil phenolics and secoiridoids. Sci. Rep..

[8] Pieroni A., Sulaiman N., Polesny Z., Sõukand R. (2022). From Şxex to Chorta: The adaptation of Maronite foraging customs to the Greek ones in Kormakitis, Northern Cyprus. Plants.

[9] Pieroni A., Morini G., Piochi M., Sulaiman N., Kalle R., Haq S.M., Devecchi A., Franceschini C., Zocchi D.M., Migliavada R. (2023). Zocchi Bitter is better: Wild greens used in the Blue Zone of Ikaria, Greece. Nutrients.

[10] Robino A., Mezzavilla M., Pirastu N., Dognini M., Tepper B.J., Gasparini P. (2014). A population-based approach to study the impact of PROP perception on food liking in populations along the Silk Road. PLoS ONE.

[11] Pieroni A., Sulaiman N., Sõukand R. (2022). Chorta (wild greens) in Central Crete: The bio-cultural heritage of a hidden and resilient ingredient of the Mediterranean diet. Biology.

[12] Molina M., Pardo-de-Santayana M., Tardío J., Sánchez-Mata M.d.C., Tardío J. (2016). Natural production and cultivation of Mediterranean wild edibles. Mediterranean Wild Edible Plants: Ethnobotany and Food Composition Tables.

[13] Martinez L., Underhill P.A., Zhivotovsky L.A., Gayden T., Moschonas N.K., Chow C.-E.T., Conti S., Mamolini E., Cavalli-Sforza L.L., Herrera R. (2007). Paleolithic Y-haplogroup heritage predominates in a Cretan highland plateau. Eur. J. Hum. Genet..

[14] Zeghichi-Hamri S., Kallithraka S., Simopoulos A., Kypriotakis Z. (2003). Nutritional composition of selected wild plants in the diet of Crete. World Rev. Nutr. Diet..

[15] Genova A. (2021). Review of A History of Crete by Chris Moorey. J. Mod. Greek Stud..

[16] Schär S., Menchetti M., Schifani E., Hinojosa J.C., Platania L., Dapporto L., Vila R. (2020). Integrative biodiversity inventory of ants from a Sicilian archipelago reveals high diversity on young volcanic islands (Hymenoptera: Formicidae). Org. Divers. Evol..

[17] Nyarota M., Chikuta O., Musundure R., Kazembe C. (2023). Indigenous Culinary Claims and Cultural Heritage Preservation: A Viewpoint. https://repository.bothouniversity.ac.bw/buir/handle/123456789/238.

[18] Duguma H.T. (2020). Wild edible plant nutritional contribution and consumer perception in Ethiopia. Int. J. Food Sci..

[19] Sulaiman N., Verner V., Polesny Z. (2023). Socioeconomic dimensions of wild food plant use during the conflict in Syria. Econ. Bot..

[20] Geraci A., Amato F., Di Noto G., Bazan G., Schicchi R. (2018). The wild taxa utilized as vegetables in Sicily (Italy): A traditional component of the Mediterranean diet. J. Ethnobiol. Ethnomed..

[21] Lentini F., Venza F. (2007). Wild food plants of popular use in Sicily. J. Ethnobiol. Ethnomed..

[22] Biscotti N., Pieroni A. (2015). The hidden Mediterranean diet: Wild greens traditionally gathered and consumed in the Gargano area, Apulia, SE Italy. Acta Soc. Bot. Pol..

[23] Hadjichambis A.C.H., Paraskeva-Hadjichambi D., Della A., Giusti E., De Pasquale C., Lenzarini C., Censorii E., Gonzales-Tejero M.R., Sanchez-Rojas C.P., Ramiro-Gutierrez J.M. (2008). Wild and semi-domesticated food plant consumption in seven circum-Mediterranean areas. Int. J. Food Sci. Nutr..

[24] Ma X., Luo D., Xiong Y., Huang C., Li G. (2024). Ethnobotanical study on ritual plants used by Hani people in Yunnan, China. J. Ethnobiol. Ethnomed..

[25] Vogiatzakis I., Mannion A.M., Pungetti G., Vogiatzakis I., Pungetti G., Mannion A.M. (2008). Introduction to the Mediterranean island landscapes. Mediterranean Island Landscapes: Natural and Cultural Approaches.

[26] Menteli V., Krigas N., Avramakis M., Turland N., Vokou D. (2019). Endemic plants of Crete in electronic trade and wildlife tourism: Current patterns and implications for conservation. J. Biol. Res.-Thessalon..

[27] Dolores M., Tongco C. (2007). Purposive sampling as a tool for informant selection. Ethnobot. Res. Appl..

[28] The ISE Code of Ethics International Society of Ethnobiology. https://www.ethnobiology.net/what-we-do/core-programs/ise-ethics-program/code-of-ethics/.

[29] Strid A. (2016). Atlas of the Aegean Flora.

[30] Rechinger K.H. (1943). Flora Aegaea; Flora der Inseln und Halbinseln des Ägäischen Meeres. Kommission bei.

[31] Turland N.J., Chilton L., Press J.R. (1995). Flora of the Cretan Area. Annotated Checklist & Atlas.

[32] (2024). Angiosperm Phylogeny Website. https://www.mobot.org/mobot/research/APweb/.

[33] El-Shazly A., Wink M. (2014). Diversity of Pyrrolizidine Alkaloids in the Boraginaceae Structures, Distribution, and Biological Properties. Diversity.

[34] Pieroni A., Sulaiman N., Prakofjewa J., Haq S.M., Zocchi D.M., Krigas N., Chryssanthopoulou V., Sõukand R. Isolated Mediterranean foraging: Wild greens in the matrifocal community of Olympos, Karpathos Island, Greece. J. Ethnobiol. Ethnomed..

[35] Marouf M., Batal M., Moledor S., Talhouk S.N. (2015). Exploring the practice of traditional wild plant collection in Lebanon. Food Cult. Soc..

[36] Pieroni A., Sõukand R., Amin H.I.M., Zahir H., Kukk T. (2018). Celebrating multi-religious co-existence in central Kurdistan: The bio-culturally diverse traditional gathering of wild greens among Yazidis, Assyrians, and Muslim Kurds. Hum. Ecol..

[37] Ali-Shtayeh M.S., Jamous R.M., Al-Shafie J.H., Wafa A.E., Kherfan F.A., Qarariah K.H., Isra S.K., Soos I.M., Musleh A.A., Isa B.A. (2008). Traditional knowledge of wild edible plants used in Palestine (Northern West Bank): A comparative study. J. Ethnobiol. Ethnomed..

[38] Pieroni A., Hovsepyan R., Manduzai A.K., Sõukand R. (2021). Wild food plants traditionally gathered in central Armenia: Archaic ingredients or future sustainable foods?. Environ. Dev. Sustain..

[39] Dop M.C., Kefi F., Karous O., Verger E.O., Bahrini A., Ghrabi Z., El Ati J., Kennedy G., Termote C. (2020). Identification and frequency of consumption of wild edible plants over a year in central Tunisia: A mixed-methods approach. Public Health Nutr..

[40] Sciandrello S., D’Agostino S., Minissale P. (2013). Vegetation analysis of the Taormina Region in Sicily: A plant landscape characterized by geomorphology variability and both ancient and recent anthropogenic influences. Lazaroa.

[41] Siebert S. (2004). Traditional agriculture and the conservation of biological diversity in Crete, Greece. Int. J. Agric. Sustain..

[42] Della A., Paraskeva-Hadjichambi D., Hadjichambis A.C. (2006). An ethnobotanical survey of wild edible plants of Paphos and Larnaca countryside of Cyprus. J. Ethnobiol. Ethnomed..

[43] Dogan Y. (2016). Wild edible plants: From the past to the future. Austin Food Sci..

[44] Risso D., Giuliani C., Antinucci M., Morini G., Garagnani P., Tofanelli S., Luiselli D. (2017). A bio-cultural approach to the study of food choice: The contribution of taste genetics, population and culture. Appetite.

[45] Tourlouki E., Matalas A.-L., Panagiotakos D. (2011). Cultural, social, and environmental influences on surviving dietary patterns of the past: A case study from the northern villages of Karpathos. Nat. Cult..

[46] Palermo M., Pellegrini N., Fogliano V. (2014). The effect of cooking on phytochemical content in vegetables: A review. J. Sci. Food Agric..

[47] Mehmood A., Zeb A. (2020). Effects of different cooking techniques on bioactive contents of leafy vegetables. Int. J. Gastron. Food Sci..

[48] Serrasolses G., Calvet-Mir L., Carrió E., D’Ambrosio U., Garnatje T., Parada M., Vallès J., Reyes-García V. (2016). A matter of taste: Local explanations for the consumption of wild food plants in the Catalan Pyrenees and the Balearic Islands. Econ. Bot..

[49] Mina G., Scariot V., Peira G., Lombardi G. (2023). Foraging practices and sustainable management of wild food resources in Europe: A systematic review. Land.

[50] Buratti S., Cappa C., Benedetti S., Giovanelli G. (2020). Influence of Cooking Conditions on Nutritional Properties and Sensory Characteristics Interpreted by E-Senses: Case-Study on Selected Vegetables. Foods.

[51] Motti R., Bonanomi G., Lanzotti V., Sacchi R. (2020). The contribution of wild edible plants to the Mediterranean diet: An ethnobotanical case study along the coast of Campania (Southern Italy). Econ. Bot..

[52] Arias-Rico J., Macías-León F.J., Alanís-García E., Cruz-Cansino N.d.S., Jaramillo-Morales O.A., Barrera-Gálvez R., Ramírez-Moreno E. (2020). Study of Edible Plants: Effects of Boiling on Nutritional, Antioxidant, and Physicochemical Properties. Foods.

[53] Razzak A., Mahjabin T., Khan M.R.M., Hossain M., Sadia U., Zzaman W. (2023). Effect of cooking methods on the nutritional quality of selected vegetables at Sylhet City. Heliyon.

[54] Lee S., Choi Y., Jeong H.S., Lee J., Sung J. (2017). Effect of different cooking methods on the content of vitamins and true retention in selected vegetables. Food Sci. Biotechnol..

[55] Bufano A. (2021). Wild Edible Plants in Italy: A Database and Its Applications in Determining Functional Compounds in Five Italian Flora Species. https://iris.unimol.it/handle/11695/105999.

[56] Lundy J., Drieu L., Orecchioni P., Meo A., Aniceti V., Fiorentino G., Primavera M., Talbot H., Molinari A., Carver M.O.H. (2023). Cuisine in transition? Organic residue analysis of domestic containers from 9th-14th century Sicily. R. Soc. Open Sci..

[57] Bajgai R., Bajgai Y., Johnson S. (2023). The presence of wild edible plants and determinants influencing their harvest, consumption, and conservation in southeastern Bhutan. PLoS ONE.

[58] Sansanelli S., Ferri M., Salinitro M., Tassoni A. (2017). Ethnobotanical survey of wild food plants traditionally collected and consumed in the Middle Agri Valley (Basilicata region, southern Italy). J. Ethnobiol. Ethnomedicine.

[59] Singh B., Sultan P., Hassan Q.P., Gairola S. (2016). Bedi, Y.S. Ethnobotany, traditional knowledge, and diversity of wild edible plants and fungi: A case study in the Bandipora district of Kashmir Himalaya, India. J. Herbs Spices Med. Plants.

[60] Diószegi J., Llanaj E., Ádány R. (2019). Genetic background of taste perception, taste preferences, and its nutritional implications: A systematic review. Front. Genet..

[61] Bachmanov A.A., Boughter J.D. (2012). Genetics of Taste Perception. eLS.

[62] Rapinski M., Raymond R., Davy D., Herrmann T., Bedell J.-P., Ka A., Odonne G., Chanteloup L., Lopez P.J., Foulquier É. (2023). Local Food Systems under Global Influence: The Case of Food, Health and Environment in Five Socio-Ecosystems. Sustainability.

[63] Zou X., Huang F., Hao L., Zhao J., Mao H., Zhang J., Ren S. (2010). The socio-economic importance of wild vegetable resources and their conservation: A case study from China. Kew Bull..

